# A Descriptive Review of the Action Mechanisms of Berberine, Quercetin and Silymarin on Insulin Resistance/Hyperinsulinemia and Cardiovascular Prevention

**DOI:** 10.3390/molecules28114491

**Published:** 2023-06-01

**Authors:** Paolo Bellavite, Serafino Fazio, Flora Affuso

**Affiliations:** 1 Pathophysiology Chair, Homeopathic Medical School of Verona, 37121 Verona, Italy; 2 Department of Internal Medicine, University of Naples Federico II, 80138 Naples, Italy; fazio0502@gmail.com; 3 Independent Researcher, 73014 Lecce, Italy

**Keywords:** insulin resistance, diabetes, food supplements, flavonoids, oxidative stress, insulin signaling, berberine, quercetin, silymarin

## Abstract

Insulin resistance (IR) and the associated hyperinsulinemia are early pathophysiological changes which, if not well treated, can lead to type 2 diabetes, endothelial dysfunction and cardiovascular disease. While diabetes care is fairly well standardized, the prevention and treatment of IR lacks a single pharmaceutical approach and many lifestyle and dietary interventions have been proposed, including a wide range of food supplements. Among the most interesting and well-known natural remedies, alkaloid berberine and the flavonol quercetin have particular relevance in the literature, while silymarin—the active principle of the *Silybum marianum* thistle—was traditionally used for lipid metabolism disorders and to sustain liver function. This review describes the major defects of insulin signaling leading to IR and the main properties of the three mentioned natural substances, their molecular targets and synergistic action mechanisms. The actions of berberine, quercetin and silymarin are partially superimposable as remedies against reactive oxygen intermediates generated by a high-lipid diet and by NADPH oxidase, which is triggered by phagocyte activation. Furthermore, these compounds inhibit the secretion of a battery of pro-inflammatory cytokines, modulate intestinal microbiota and are especially able to control the various disorders of the insulin receptor and post-receptor signaling systems. Although most of the evidence on the effects of berberine, quercetin and silymarin in modulating insulin resistance and preventing cardiovascular disease derive from experimental studies on animals, the amount of pre-clinical knowledge strongly suggests the need to investigate the therapeutic potential of these substances in human pathology.

## 1. Introduction

Over the last decades, great progress has been made in the prevention and treatment of cardiovascular diseases, which, in Italy, has led to a drop in cardiovascular mortality of about 53% between 1980 and 2010. However, data updated by the Central Statistical Office of the Istituto Superiore di Sanità (ISS) still indicate high mortality from cardiovascular diseases, accounting for about 39% of total deaths in Italy [1]. In the member countries of the European Union, cardiovascular diseases currently claim 2 million lives each year and account for 42% of total deaths [2]. Therefore, although much has been done, a lot more remains to be achieved in terms of the prevention and treatment of cardiovascular risk factors. While significant progress has been made in treating dyslipidemias, diabetes mellitus and hypertension, little is being done in the field of early screening and treatment of insulin resistance/hyperinsulinaemia (IR/Hyperin), as independent risk factors for cardiovascular diseases [3,4].

Insulin resistance (IR) is a silent pandemic and a serious public health concern: it has been reported that between 15.5 and 51% of adults in highly developed countries are affected [5]. IR not only affects obese individuals but normal or underweight people as well, since several different mechanisms underlie the pathogenesis of this disorder. Chronic inflammation, sedentariness, alterations in intestinal microbiota and, above all, genetic factors can be quite frequent [5,6,7]. Although overweight and obesity are commonly associated with diabetes, a Kaiser Permanente study found that this connection differs widely according to race or ethnicity. Indeed, normal-weight Hawaiians and Pacific Islanders were three times more likely to have diabetes than normal-weight white people. In fact, diabetes prevalence with normal BMI was 18% for Hawaiians/Pacific Islanders versus just 5% for Whites. Prevalence was also higher among Blacks, (13.5%), Hispanics (12.9%), Asians (10.1%) and American Indians/Alaskan natives (9.6%) [6].

In many cases, IR can be asymptomatic or paucisymptomatic and the affected subjects, when treated, are mostly addressed to lifestyle modification with frequent non-adherence and treatment failure. This review aims to evaluate the need to counter the underlying mechanisms and not the consequences of IR; furthermore, in the absence of an authorized drug therapy, it is mandatory to intervene to avoid future complications.

There are many natural substances in the scientific literature that show the beneficial effects on IR and Hyperin, although mainly in basic research studies. Among the various natural substances that have shown efficacy against IR and Hyperin, we have decided to carry out a review of the literature on the effects on these conditions of the isoquinoline alkaloid berberine (Bbr), the flavonol quercetin (Qtn) and silymarin (Smn), a mixture of flavonolignans extracted from the blessed milk thistle (*Silybum marianum*). These substances were chosen for various reasons: because we have good knowledge and experience with them, having studied them extensively in the past [8,9,10,11,12,13,14]; because they appear in the largest amount of the scientific literature on this topic (see for examples Table 1); because they may have synergistic mechanisms that can lead to an increase in efficacy, as we explore in this review; and because, to our knowledge, this combination has never been investigated before. Moreover, in the case of silymarin, an additional mechanism acting on the absorption of the other substances has also been demonstrated and will be described in more detail later.

This is a descriptive review of the pathophysiologic mechanisms by which IR/Hyperin should be considered a risk factor for cardiovascular diseases and of the mechanisms of action and potential beneficial effects of these natural substances on the early disorders of glucose metabolism and, consequently, on the cardiovascular system. The scientific literature used for this review was found in PubMed, Scopus, Science Direct, using the following keywords: insulin resistance, hyperinsulinemia, berberine, quercetin, silymarin, metabolism, cardiovascular disease prevention. Although there is a considerable number of studies showing the positive effects of these natural substances in the treatment of dyslipidemia as well, the analysis of this issue will not be addressed in this review both for reasons of space and because we wish to emphasize the effects of Bbr, Qtn and Smn on IR/Hyperin.

## 2. Insulin Resistance and Hyperinsulinemia: Pathophysiological Mechanisms of Cardiovascular Damage

IR is a pathological condition characterized by a decrease in sensitivity and responsiveness to the metabolic actions of insulin, so that at a given concentration of the hormone there is clearly less biological effect than expected. For this reason, increased levels of insulin are essential to achieve normal glucose tolerance, and hyperinsulinemia is one of the main features of IR states [15].

IR represents a pivotal mechanism of type 2 diabetes, hypertension and cardiovascular diseases. While it may be easily recognized in the context of the metabolic syndrome and polycystic ovary syndrome, in other cases a diagnosis could be difficult in normal or underweight individuals. Many studies have demonstrated that IR is the earliest abnormality in the natural history of type 2 diabetes, due to defects in both the action of insulin (IR) and its secretion (beta cell dysfunction). Some of these studies have also shown that hyperinsulinemia, resulting from IR, anticipates the development of type 2 diabetes even up to 10–15 years [7,15].

Increased circulating insulin concentrations can be considered a highly suggestive parameter of IR. The gold standard technique for diagnosing IR is the euglycemic hyperinsulinemic clamp, which is complex, expensive and a rather invasive procedure, and therefore, cannot be used for mass screening but only scientific purposes [16,17].

Simpler substitute indices have been created for clinical screening: the Homeostasis Model Assessment of IR Index [HOMA:IR = fasting insulin (µIU/mL) × fasting glycemia (mmol/L)/22.5] and the triglyceride-glucose index (TyG index = Logarithm of the product between triglycerides and fasting blood glucose/2) can be considered the best surrogate IR markers, since they correlate with values derived from the euglycemic-hyper insulinemic clamp. A HOMA-IR value of between 0.23 and 2.5 can be considered normal in an adult population, while the cut-off for the TyG index is 4.5 [17,18,19].

**Figure 1:**
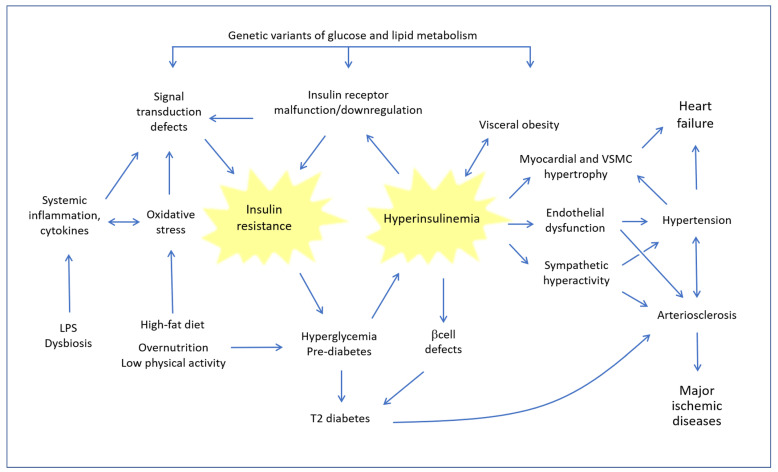
Dynamic relationships between insulin resistance and hyperinsulinemia and their consequences on the cardiovascular system. LPS: Lipopolysaccharide; VSMC: Vascular Smooth Muscle Cells.

Figure 1 summarizes the main relationships between IR and cardiovascular diseases.

At the heart of the problem is a vicious circuit, whereby the chronic tendency to increase blood sugar, caused by lifestyle and a high-fat diet (HFD), and conditioned by genetic factors, leads to hyperglycemia, which in turn induces an increase in the compensatory synthesis of insulin. However, when the situation persists and consolidates, in the presence of concomitant disturbing factors such as oxidative stress and systemic inflammation, hyperinsulinemia leads to a progressive defect of the insulin receptor (INSR) and of downstream signal transduction systems involved in IR. The pathogenic circle is closed because IR, in turn, increases the tendency to hyperglycemia. As the years go by, pancreatic beta cells gradually become deficient and the pathophysiology of frank type 2 diabetes occurs. Even before overt diabetes sets in, this type of imbalance has profound consequences on the cardiovascular system for the following reasons: (a) a reciprocal strengthening between hyperinsulinemia and obesity, with the well-known consequences of the latter; (b) the direct hypertensive effect of insulin, which stimulates the sympathetic system and increases renal sodium reabsorption; (c) a direct action on the growth of myocardial muscle cells and eventually cardiac hypertrophy; and (d) endothelial dysfunction and increased thickness of the arterial wall, which contribute to the development of arteriosclerosis. All these pathological changes, adding on to the pathogenic action of hyperglycemia and protein glycation, Advanced Glycation End Products (AGE) and eventually to the other classic atherogenic factors (smoke, hyperhomocysteinemia, dyslipidemia, etc.), progressively result in the most serious ischemic organ pathologies.

Left ventricular hypertrophy has been observed in IR conditions, even in individuals who had not yet developed arterial hypertension. Moreover, it must be emphasized that in IR/Hyperin conditions an alteration occurs in the inflammation mechanisms due to a chronic stimulation of pro-inflammatory pathways. Particularly in obese and overweight individuals with IR, adipose cells hypersecrete several adipocytokines, such as tumor necrosis factor alpha (TNF-ɑ), resistin and interleukin-6 (IL-6), which chronically promote vascular inflammation by stimulating the development of atherosclerosis and aggravating endothelial dysfunction [20,21].

For these reasons, many studies are under way to understand the mechanisms of IR and to find drugs or food supplements capable of slowing down or interrupting the pathological dynamics described.

### 2.1. Insulin Resistance Mechanisms

Insulin is a polypeptide hormone composed of 51 amino acids, secreted by the beta cells of the pancreas, which intervenes in many important biological processes with an action that is mediated by a transmembrane tyrosine kinase receptor. It intervenes in an important way in glucose homeostasis and metabolism, as well as in cell growth. The binding of the hormone to INSR in the various target tissues leads to the activation of complex insulin-signaling transduction pathways, and therefore, it increases glucose uptake in fat and muscle cells and reduces glucose synthesis by the liver, so it is strongly involved in maintaining glucose homeostasis [22]. Protein tyrosine kinases (PTKs) catalyze the phosphorylation of tyrosine, a key reversible post-translational mechanism that is required for metabolic homeostasis, and the regulation of cell growth and differentiation. This covalent modification is a reversible mechanism of protein regulation in which PTKs catalyze phosphorylation, while protein tyrosine phosphatases (PTPs) are responsible for removing tyrosine-bound phosphate groups. The activities of PTKs and PTPs are coordinated, so that PTKs amplify the signal response, while PTPs regulate the speed and duration of the response. Altered regulation of PTKs or PTPs have been found in many types of cancer, and changes in tyrosine phosphorylation are associated with metabolic disorders. For this reason, PTPs have been investigated as pharmacological targets for their role in slowing down the negative evolution of metabolic and cardiovascular damage.

The INSR is a transmembrane protein tyrosine kinase that phosphorylates itself and target substrates, such as IRS-1, by binding to insulin. These phosphorylation events allow for the recruitment and activation of various signaling pathways, including PI3K/Akt and Ras/mitogen-activated protein kinase (MAPK) pathways. Two important pathways, which underlie the actions of insulin, have been identified: (1) the inositide phosphate-3-kinase (PI3K) dependent pathway, which predominantly mediates the metabolic actions of the hormone by regulating glucose metabolism in muscle, adipose and liver tissues, and regulating nitric oxide (NO) formation by endothelial cells and vascular smooth muscle cells (VSMCs) [23]; and (2) the mitogen-activated protein kinase (MAPK) dependent pathway, which mediates primarily the mitogenic and proliferative action, the induction of endothelial cells to secrete increased amounts of endothelin-1 (ET-1) and the increased expression of adhesion molecules on vascular endothelium [24]. Under normal conditions, these pathways, in equilibrium, contribute to maintaining vascular homeostasis. In fact, the former (NO-dependent) causes vasodilation and a reduction in vascular resistance with an increase in tissue blood flow and stimulation of capillary recruitment, while the latter (ET-1-dependent) causes vasoconstriction with activation of the sympathetic system, thus exerting a hypertensive effect and accelerating the development of atherosclerosis [25,26].

Plenty of evidence is now available supporting the role of the protein kinase phosphatase receptor (RPTPs) in the signaling and secretion of insulin, and consequently, in IR conditions up to type 2 diabetes mellitus. Therefore, the use of molecules that alter the ligand binding to the extracellular domain of RPTPs, which regulate RPTP activity, could open new perspectives in the treatment of many metabolic and cardiovascular diseases [27].

5′-Adenosin Mono Phosphate-activated protein kinase (AMPK) acts as a cellular energy state sensor that is activated by increases of the AMP/ATP ratio caused by metabolic stresses, such as reduced availability of glucose or oxygen in the tissues. The relationships between insulin and AMPK are very complex and still not fully clarified, since they can differ in the different tissues. However, in skeletal muscle both insulin and AMPK go in the same direction, particularly regarding the glycemic level regulation processes. In fact, in this case, both increase glucose uptake, increasing transmembrane translocation of glucose transporter type 4 (GLUT4) [28]. AMPK phosphorylates and activates the INSR and promotes energy conservation by stimulating the entrance of glucose in the skeletal muscle tissue [29].

**Figure 2:**
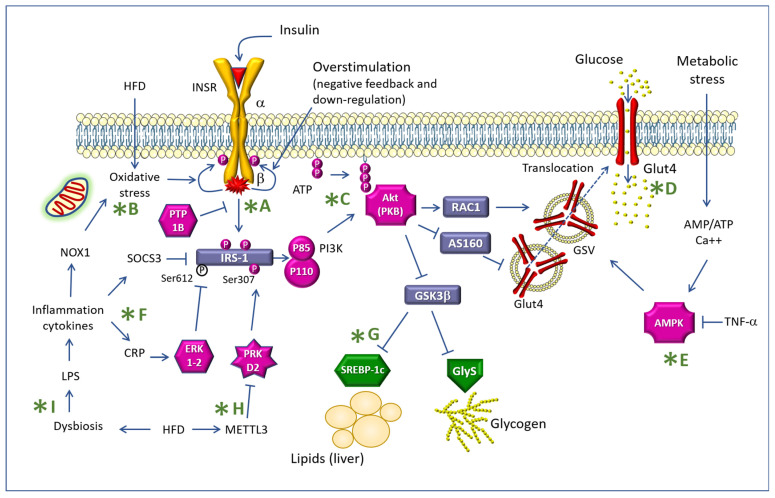
Mechanisms of insulin resistance indicating some targets of the action of the polyphenols described in the text (green asterisks). Legend: INSR: Insulin receptor; IRS-1: Insulin receptor substrate-1; PTP1B: Protein tyrosine phosphatase 1B; Akt: Ak mouse thymoma; PKB: Protein Kinase B; HFD: high-fat diet; NOX1: NADPH oxidase-1; SOCS3: suppressor of cytokine signaling 3; CRP: C-reactive protein; LPS: lipopolysaccharide; PI3K: phosphatidyl-inositol 3 kinase; METTL3: Methyltransferase Like 3; RAC1: Rac family small GTPase 1; GSK3β: Glycogen synthase kinase-3β; ERK: extracellular signal-regulated kinases; PRKD2: Protein Kinase D2; SREBP-1c: sterol regulatory element binding transcription factor 1; GlyS: glycogen synthetase; GSV: GLUT4 storage vesicle; AMPK: AMP-activated protein kinase; TNF-α: Tumor necrosis factor-α; The letters after the green asterisk are referred in the text of the manuscript.

Figure 2 summarizes the main transduction pathways of insulin signals in the cell, indicating the steps where a mechanistic contribution of the polyphenols described in this text was reported.

Skeletal muscle is an important site for glucose metabolism and IR. Skeletal muscle IR can be attributed to defects at the more proximal levels of insulin signaling: INSR, INSR substrate 1 (IRS-1), phosphatidylinositol 3-kinase (PI3K) and Akt activity, also named as protein kinase B [30]. The biochemical pathway IRS-1/PI3K/Akt/glycogen synthase kinase-3β (GSK-3β) plays a critical role in mediating the metabolism of insulin by promoting the translocation of GLUT4 to the membrane and the uptake of the glucose synthesis [31] (see Figure 2). However, overeating and sedentary modern lifestyles disrupt this system and can cause IR conditions, including metabolic syndrome, obesity, type 2 diabetes mellitus (T2DM) and cardiovascular disease [32]. AMPK is another activator of GLUT4, albeit in an insulin-independent manner [33].

The above indicates that, in normality, there is a close association between the metabolic and hemodynamic actions of insulin. In IR conditions, the PI3k-dependent metabolic pathways are specifically impaired, whereas MAPK-dependent pathways are generally less involved. This is why hyperinsulinemia, which is typically associated with IR in the attempt to maintain normal glucose levels, ends up hyper-stimulating the MAPK pathway, producing an imbalance of the two pathways with an increase in the MAPK effects of insulin, in particular overproduction of ET-1 and reduced NO production, resulting in vasoconstriction and endothelial dysfunction. Hyperinsulinemia can lead to arterial hypertension not only via the increase in ET-1 secretion and sympathetic tone, but also because it can lead to anti-natriuretic effects. At the renal level, in fact, the hormone stimulates sodium reabsorption in the distal tubule [34]. Moreover, hyperinsulinemia, through its mitogenic and proliferative effects, can lead to VSMC proliferation and myocardial hypertrophy [35,36].

Insulin is also an important growth factor, although weaker than insulin-like growth factor-1, vascular endothelial growth factor, platelet-derived growth factor, and epidermal growth factor. It stimulates cell growth, cell division and inhibits apoptosis. Growth factors to stimulate mitogenesis must activate the pathway of Ras-Raf-Map kinase. Ras proteins are activated by binding triphosphates guanosine (GTP), a process that is activated by Sos guanine nucleotide exchange factor [37]. Hyperinsulinemia in IR conditions stimulates phosphorylation and activation of farnesyltransferase (FTase), ubiquitous enzyme that farnesylates the protein Ras. The increased availability of Ras, farnesylated at the level of cytoplasmic membranes, increases the cellular response to various growth factors. Insulin stimulation of FTase is one important mechanism underlying the mitogenic and pro-atherosclerotic effects of the hormone [38].

In recent years, it has been reported that dipeptidyl peptidase-4 (DPP4) is elevated in IR/Hyperin conditions [39]. DPP4 is a serine protease that cuts peptides with the N-terminal complex and is widely present in many cell types, such as endothelial cells, fibroblasts and lymphocytes, and is mostly found in its dimer form in the cell membranes. Catalytically active DPP4 is released from cell membranes into plasma in a circulating soluble form. It has many substrates for its pleiotropic activities and drugs that inhibit DPP4 activity have been widely considered for their important role in glucose metabolism, as they prevent the degradation of glucagon-like peptide-1 (GLP-1) and glucose insulinotropic peptide (GIP). The actions of DPP4 mimic the atherogenic actions of hyperinsulinemia, and DPP4 inhibition in pro-atherosclerotic preclinical models resulted in a reduction of inflammatory and oxidative stress mechanisms, improved endothelial dysfunction, and reduced the development of atherosclerosis [40,41,42]. Thus, DPP4 could represent a key link between IR/Hyperin conditions and the development of atherosclerosis. Despite the strong evidence in preclinical studies implicating both hyperinsulinemia and DPP4 activity in the pathogenesis of atherosclerosis, multiple large clinical trials of DPP4 inhibitors have failed to demonstrate a reduction in cardiovascular outcomes in individuals with type 2 diabetes mellitus [43].

### 2.2. Hyperinsulinaemia as a Cardiovascular Risk Factor

Recently, it has also been hypothesized that hyperinsulinemia present in the IR condition of obese subjects who have not yet developed diabetes may be due to altered insulin clearance, while insulin secretion remained unmodified [44]. However, whatever the cause—increased secretion, reduced clearance or the sum of both—hyperinsulinemia remains the major marker of IR conditions with all its negative implications.

This set of premises suggests that IR/Hyperin, besides inevitably evolving toward diabetes without adequate correction, must be considered a cardiovascular risk factor and therefore be taken into account in screening and treatment to slow down the evolution toward diabetes and cardiovascular complications. It is well known, in fact, that most individuals who have developed overt type 2 diabetes after years of IR and hyperinsulinemia already have, at the time of diagnosis, evident cardiovascular alterations—and in fact, by guidelines, they are subjected to more severe degree of cardiovascular prevention and treatment lines [45].

Patients diagnosed with IR are mostly instructed to change their lifestyle but are generally not prescribed drugs with the indication of reducing IR. This is also because, in the initial phase, IR and hyperinsulinemia may be completely asymptomatic, and therefore, any pharmacological treatment would be poorly accepted by patients. Unfortunately, as widely verified in everyday clinical practice, few patients take action to correct the errors in their daily life routines. These people could benefit from taking some natural substances that have been researched to verify their positive action in reducing IR and insulin levels, with the aim of slowing down evolution toward overt diabetes, but above all, to prevent cardiovascular damage. Among the most studied for this purpose, based on their action mechanisms, Bbr, Qtn and Smn appear to be the most promising.

**Figure 3:**
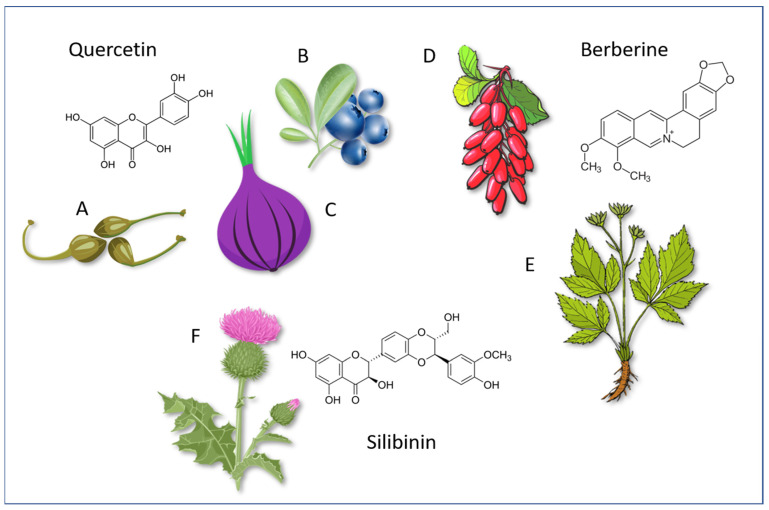
Molecular structures of Quercetin, Berberine and Silibinin and some foods and plants that contain particularly important doses. A: Capes (*Capparis spinosa*); B: Blueberry (various plants of *Vaccinium* genus); C: Red onions (*Allium cepa*); D: Barberry (*Berberis vulgaris*) berries; E: *Coptis chinensis* (used with its dried roots); F: *Silybum marianum*.

Figure 3 shows the molecular structures of these compounds (for Silymarin the most important polyphenol Silibinin is shown) and a scheme of their possible synergism in counteracting IR and its consequences.

## 3. Effects of Berberine on Insulin Resistance/Hyperinsulinemia and Cardiovascular Changes

Bbr is a natural alkaloid isolated from Chinese goldthread (*Coptis chinensis*) and present in several plants, including Barberry (*Berberis vulgaris*) edible berries, Oregon Grape (*Mahonia aquifolium*) and Goldenseal (*Hydrastis canadensis*). These and other plants have been used for more than 2000 years in both Chinese and Ayurvedic medicine. Plants with a high Bbr content, such as *Fibraurea tinctoria*, have recently been proposed as antidiabetic for their antioxidant properties [46]. Although Bbr has been used for years in Asian countries for its anti-microbial effects, particularly for intestinal infections and diarrhea, and for its beneficial effects on diabetes mellitus, it has only come to the forefront in Western countries in recent decades because of its positive effects in the treatment of metabolic and cardiovascular diseases. Its beneficial activity on glucose and lipid metabolism, endothelial function and the cardiovascular system has been studied extensively in recent decades with very appreciable results in both animal studies and clinical studies in humans [47].

### 3.1. Effects of Berberine on Glucose Metabolism

Bbr has been used for many years in China as an oral hypoglycemic in the treatment of type 2 diabetes mellitus and its effects on glucose metabolism are sufficiently well known. Its hypoglycemic action has been verified in subjects with type 2 diabetes and was compared to that of metformin (Met) in a randomized study. Subjects with newly diagnosed type 2 diabetes mellitus were randomized to receive Bbr or Met in a 3-month trial, and the hypoglycemic effects proved similar, with a significant (*p* < 0.01) reduction in glycated hemoglobin and postprandial glycemia and a significant reduction in triglycerides. Moreover, in the same study, a group of 48 patients with poorly controlled type 2 diabetes received a Bbr supplement for 3 months and their glycated hemoglobin levels decreased from 8.1 ± 0.2% to 7.3 ± 0.3% (*p* < 0.001), with the HOMA-IR significantly dropping as well (*p* < 0.001) [48]. The reduction of fasting and postprandial blood sugar was also confirmed by a double-blind placebo-controlled study in a group of 146 patients with type 2 diabetes and dyslipidemia. These results were also associated with a slight reduction in postprandial insulin and body weight [49].

A prospective, double-blind, randomized, placebo-controlled study, conducted in 64 patients with metabolic syndrome, showed a significant reduction (*p* < 0.001) of HOMA-IR after 18 weeks of treatment with a nutraceutical combination containing Bbr [9].

Several basic studies have investigated how Bbr exerts its positive effects on glucose metabolism and insulin sensitivity. Bbr also increases insulin sensitivity because it increases INSR gene expression, in a dose and time dependent manner (see *A in Figure 2). By increasing receptors, Bbr improves glucose consumption in the presence of insulin. This mechanism was also confirmed in a subsequent study carried out on patients with type 2 diabetes mellitus by the group of Zhang H. [50].

In 2006, a group of researchers [51] paved the way for further investigation of the mechanisms of Bbr in the treatment of diabetes, obesity and IR by conducting in vitro and in vivo experiments. They found that Bbr stimulated AMPK, an enzyme protein that plays a major role in regulating the whole body’s energy homeostasis (*E in Figure 2). Indeed, the administration of Bbr to db/db mice resulted in a significant reduction in their weight associated with a significant reduction in fasting glycaemia, with an improvement in glucose tolerance [51]. Similar effects were also reported in a study on high-fat-fed Wistar rats, in which Bbr resulted in reduced triglycerides and body weight and improved insulin action compared to chow-fed rats. In this case, Bbr was found to down-regulate the expression of genes involved in lipogenesis and up-regulate those involved in energy expenditure in adipose tissue and muscle [51]. Subsequently, it was reported that Bbr increases glucose uptake in 3T3-L1 preadipocytes by increasing the expression of the GLUT-1 gene, unlike insulin, which acts by promoting the expression of GLUT-4 on the cell surface by activating PI3K (*C in Figure 2) [52,53]. These effects of Bbr were found to be mediated also by AMPK, resulting in an improvement in energy production and a reduction of energy storage [54]. These data were subsequently confirmed by other studies. In obese hyperinsulinemic rats, Bbr resulted in a significant reduction in blood glucose, circulating insulin levels, and weight. Bbr has been shown to suppress the proliferation and differentiation of 3T3-L1 pre adipocytes and to reduce the accumulation of lipid drops in the process of differentiation, working on multiple molecular targets as a gamma peroxisome proliferator-activated receptor (PPAR-γ) inhibitor. For this reason, unlike other substances, it can also produce weight reduction [55].

In addition to PPAR-γ, Bbr alone or in synergy with other phytocompounds has many other molecular targets that regulate glycidic metabolism [56]. Furthermore, insulin tolerance studies showed a clear improvement in IR status and it was also observed that Bbr acutely reduced, via the AMPK signaling pathway, glucose-stimulated insulin secretion by pancreas beta cells isolated from rats [57]. In cultured human liver cells, as well as in rat skeletal muscle cells, Bbr was found to increase INSR messenger RNA (*A in Figure 2) and to improve cellular glucose consumption in the presence of insulin. Bbr increased INSR gene expression through a protein-kinase-C-dependent activation of the promoter. In rats with type 2 diabetes mellitus, Bbr decreased fasting blood glucose and serum insulin levels and increased insulin sensitivity. This did not happen in rats with type 1 diabetes, and therefore, in the absence of insulin [58].

The protein tyrosine phosphatase PTP1B is particularly important in the regulation of the receptor and is a physiological regulator of glucose homeostasis [59]. PTP1B dephosphorylates INSR and IRS-1 by reducing the overall signaling pathway and is therefore involved in IR [60] (*A in Figure 2). Inhibition of this phosphatase is recognized as a promising antidiabetic activity of many herbal compounds, including epigallocatechin 3-gallate, Qtn, berberine, rutin, hesperidin [61,62,63,64,65,66,67]. In 2005, Bustanji et al. reported that Bbr inhibits human protein tyrosine phosphatase 1B (h-PTP1B) by binding the pocket of h-PTP1B in a low energy orientation, and this may be one of the mechanisms by which Bbr has anti-hyperglycemic action [68], since inhibition of phosphatase leads to increased phosphorylation of IRS-1 and INSR itself [61]. The cAMP response element-binding protein (CREB) is a cellular transcription factor that binds to certain DNA sequences called cAMP response elements (CRE), increasing or decreasing the transcription of downstream genes. Bbr accelerates the cellular degradation of cAMP by activating the cAMP degrading enzyme (DPE), and consequently, blocks the hepatic glucagon pathway by downregulating the phosphorilation of CREB, and therefore, the gluconeogenesis genes. For this reason, Bbr plays a fundamental role as regulator of gluconeogenesis in diabetes [69].

Steatohepatitis is an important consequence of IR/Hyperin, itself aggravated by steatohepatitis. In a recent study, Shu et al. have shown that Bbr alleviates nonalcoholic steatohepatitis in mice by modulating the interaction of the gut microbiota and bile acid metabolism, and by activating the intestinal Farnesoid X Re-ceptor (FXR), which is a bile acid receptor which thus regulates metabolism of bile acids and the gut microbiota [70] (see also chapter 7).

### 3.2. Effects of Berberine on the Cardiovascular System

Diseases linked to atherosclerosis are the leading causes of death in the Western world. About half of the over-45 population has atherosclerosis without knowing it. All the arteries in the body can be affected, becoming more dangerous when the arteries of vital organs are affected. Endothelial cell dysfunction, oxidative stress and chronic inflammation play important roles, resulting in a vicious circle in the pathogenesis and worsening of atherosclerosis [71].

It is well known that endothelial dysfunction (ED) appears early on in the development of the atherosclerotic process (Figure 1). ED is characterized by nicotinamide-adenine-dinucleotide-phosphate-oxidase (NADPH oxidase) activation, endothelial-nitric-oxide-synthase (eNOS) uncoupling, increased expression of ET-1, increased production of adhesion molecules [72]. This altered endothelial function contributes to initiating and advancing the atherosclerotic process.

Many studies report an effective action of Bbr against endothelial dysfunction and the growth of vascular smooth muscle cells and myocardiocytes. Flow-mediated dilation (FMD) is an adequate early marker of vascular dysfunction. The positive effects of Bbr on FMD were shown in a clinical, double-blind, placebo-controlled trial in which Bbr was administered in combination with other natural substances in a group of patients with IR. This treatment produced a significant increase of FMD, showing a clear improvement of endothelial dysfunction [8]. More recently, a multicenter, randomized, double-blind, placebo-controlled trial of 158 patients, with insulin-resistance and left ventricular hypertrophy (LVH), treated with a nutraceutical combination containing Bbr or a placebo for 24 weeks, showed that the treatment with the nutraceutical combination was associated with a significant reduction of left ventricular mass and LVH, indicating that these substances could represent an effective strategy to reduce cardiovascular risk [73]. A clinical study has also shown an anti-inflammatory action of the substance. An amount of 130 patients with acute coronary syndrome undergoing percutaneous coronary intervention (PCI) were recruited in a study in which 61 patients were treated with Bbr, in addition to standard treatment, whereas the remaining 69 received standard therapy alone. In the Bbr-treated group, matrix-metalloproteinase-9, intracellular adhesion molecule-1, vascular cell adhesion molecule-1, C-reactive protein, IL-6 and monocyte chemoattractant protein –1 were all significantly lower in the group treated with the Bbr plus standard therapy than in the group treated with the standard therapy alone [74], showing an improvement of inflammatory parameters after 1-month treatment with Bbr in PCI patients. Furthermore, a recent meta-analysis of randomized controlled trials, aimed to systematically evaluate the effects of Bbr treatment on inflammatory markers in IR/Hyperin conditions, concluded that the use of Bbr significantly decreased inflammatory markers in these conditions [75].

As already seen in the chapter on the effects of Bbr on glucose metabolism and IR, this substance has widely shown its capacity to reduce insulin plasma levels. Therefore, since insulin is a growth factor, its reduction by Bbr could by itself explain a beneficial impact of this substance on vascular and myocardial growth. However, there is substantial scientific literature that demonstrates the consistent positive effects of Bbr on the development of atherosclerosis and the prevention of cardiovascular diseases. Using network pharmacology to study the interactions between Bbr and atherosclerosis, many potential targets related to this issue were identified. Among the most active, MAPK and PI3K–Akt signaling pathways (*C and *E in Figure 2), provide clear evidence for the mechanisms of positive effects of Bbr on atherosclerosis [76].

An in vitro investigation by Ko et al. [77] showed that Bbr has both vasorelaxant and antiproliferative actions. The mechanisms determining this vasorelaxant effect were clarified later by experiments performed on cultures of endothelial cells derived from rats. The vasodilatory effects of Bbr were due to increased eNOS activity, resulting in increased NO production via activation of the AMPK cascade. In addition, Bbr counteracts other adverse effects of hyperglycemia and hyperinsulinemia on vascular homeostasis, including inhibition of intracellular reactive species accumulation (*B in Figure 2), apoptosis and inflammation (*F in Figure 2) [78]. In Sprague Dawley, rats with suprarenal aortic constriction received an eight-week treatment with Bbr, which determined cardiac growth inhibition. In particular, left ventricular hypertrophy was reduced with a clear improvement of contractility and relaxation indices [79]. Additionally, in rats with experimental left ventricular hypertrophy, Bbr was shown to reduce plasma levels of catecholamines and adrenaline was reduced both in plasma and in left ventricular muscle tissue [80].

Extensive experimental basic experiences support a beneficial role of Bbr in the control of oxidative stress and chronic inflammation. The antioxidant activity of Bbr has been verified in in vitro studies and animal models. These studies have shown that Bbr beneficially modified the levels of antioxidant enzymes and reduced those of oxidative stress makers. It counteracts oxidative stress by eliminating useless and harmful free radicals [74,81]. An in vitro study has demonstrated, in a dimethyl sulfoxide (DMSO) alkaline environment, the ability of Bbr to scavenge superoxide free radicals. Oxidative stress is also reduced by Bbr by inhibiting the expression of NADPH oxidase [82], which has a key role in the origin of reactive oxygen species (ROS) in hyperglycemia and IR conditions (*B in Figure 2) [83]. The inhibitory effect on the NADPH oxidase expression by Bbr could suppress the formation of ROS and prevent the damages produced over time by type 2 diabetes mellitus and IR/Hyperin conditions [84].

In obesity and other IR conditions, there is an increased secretion of proinflammatory cytokines. Recently, the role that Bbr could have on the gastrointestinal microbiota (*I in Figure 2) has also been discussed as a mechanism for its beneficial effects on IR/Hyperin and cardiovascular disease prevention, although much has yet to be clarified [85].

Adverse effects of Bbr are modest, predominantly present in the gastrointestinal tract with a treatment with one gram a day or more. They appear almost exclusively within the first 4 weeks of treatment and disappear, in most cases, after a week, by reducing the dose of Bbr below 1 g a day. The principal events include diarrhea (10.3%), constipation (6.9%), flatulence (19%) and abdominal pain (3.4%) [48].

In short, Bbr may improve IR/Hyperin through multiple mechanisms, including activation of AMPK, inhibition of inflammation, reduction of oxidative stress, modulation of gut microbiota, regulation of glucose metabolism and activation of insulin signaling pathways. Furthermore, it may have beneficial effects on cardiovascular system by its action of reducing MAPK activity, regulating vascular smooth cell activity, improving endothelial function and reducing inflammation. Furthermore, the positive effects of lipid metabolism regulation are also very important, although this is not a topic of this review. Given the multiple positive data that support the beneficial activity of Bbr on IR/Hyperin and cardiovascular disease prevention, it would be extremely important to carry out large-scale clinical randomized, double-blind and placebo-controlled trials on a large sample of patients, to verify once and for all the effectiveness of this substance according to the well-known standards of evidence-based medicine.

## 4. Quercetin as a Modulator of Insulin Resistance

Qtn is a flavonoid, belonging to the flavonol group. Qtn (molecular formula C15H10O7, mass: 302.236 g/mol, density: 1.8 g/cm³) is the aglycone component of various glycosides, including rutin and quercitrin, and is found in abundant quantities in the diet because it is present in many edible vegetables, such as red onions, capers, broccoli, chicory excel, lettuce and apples. It is also found in non-edible vegetables, such as horse chestnuts, calendula, hawthorn, chamomile, St. John’s wort (*Hypericum perforatum*) and *Ginkgo biloba*. The fava d’Anta bean (*Dimorphandra mollis*) is particularly rich in Qtn, so much so that it is also used as an economically convenient raw material for its purification.

Known for its antioxidant and anti-inflammatory properties, Qtn is proposed as a dietary supplement in antiaging and immunostimulant formulations. Numerous studies demonstrate its potential usefulness in the treatment and prevention of various morbid conditions: allergies, atherosclerosis, arthritis, Alzheimer’s disease, psoriasis, lupus and many of the pathologies linked to aging.

Qtn inhibits numerous steps leading to the release of histamine and the production of pro-inflammatory prostaglandins and leukotrienes, as well as the enzymes 5-lipoxygenase and phospholipase A2. At the same time, it exerts a powerful direct and indirect antioxidant action, protecting the activity of the endogenous antioxidant enzymatic systems: catalase, superoxide dismutase, glutathione peroxidase and glutathione reductase.

Preventing or treating IR means preventing diabetes and its complications (Figure 1). IR is linked to metabolic syndrome, endothelial dysfunction and vascular disease in reciprocal and synergistic relationships [86,87,88,89]. Indeed, subjects with metabolic syndrome have significantly higher levels of insulin, endothelin and pro-thrombotic markers and low levels of nitric oxide [90]. It has been shown that individuals with these characteristics are also commonly prone to chronic inflammatory states and oxidative stress phenomena, probably due to mitochondrial nutrient metabolism disturbance [91].

In this context, it is interesting that good results have been obtained in clinical studies with the integration of various polyphenols [92,93,94,95], including Qtn [88,96,97]. A meta-analysis [98] identified nine studies on this topic, which overall demonstrated that Qtn supplementation did not affect fasting blood glucose or IR. However, in subgroup analyses, Qtn supplementation slightly—but significantly—reduced fasting glucose in studies of 8-week duration and using Qtn in doses equal to or greater than 500 mg/day. Better effects were found in individuals <45 years of age. The supplementation of Qtn nutrition on blood pressure and endothelial function among patients with metabolic syndrome was studied with a meta-analysis [93]. The authors found a significant reduction in systolic but not diastolic blood pressure.

Qtn has been detected in plasma, in pharmacologically active doses, after consumption of food or supplements and interacts with many molecular targets in the gut, skeletal muscle, adipose tissue and liver to control glucose homeostasis [99]. Experimental evidence, obtained on in vitro animal and cellular models, of the actions of Qtn to counteract IR and consequently regulate glucose and lipid metabolism, is summarized in Table 2, in chronological order.

Based on the IR mechanisms already described and the experimental evidence, it is clear that the action mechanisms of Qtn are pleiotropic [119,120]. Qtn affects signaling pathways involved in IR and the pathogenesis of type 2 diabetes, such as nuclear factor erythroid 2-related factor2 (Nrf2) (involved in antioxidant systems), nuclear factor kB (NF-kB) (inflammatory cytokine transcription factor), AMPK and Akt [121].

Schematically, it is possible to group the actions of Qtn according to three different strands: antioxidant action, regulation of protein phosphorylation chains and anti-inflammatory action.

### 4.1. Antioxidant Action and Inhibition of NADPH Oxidase

Oxidative stress contributes to IR in various ways. High-fat diets increase mitochondrial H_2_O_2_ production and cause a reduction in the glutathione (GSH)/glutathione disulfide (GSSG) ratio [122]. Since the activity of many protein kinases and phosphatases is regulated by the redox state of cysteine thiols, a more oxidized cellular environment may favor the serine/threonine phosphorylation events that characterize the negative feedback of insulin action. Mitochondrial H_2_O_2_ clearance, either by pharmacological means or by transgenic expression of catalase in mitochondria (MCAT mice), protects against HFD-induced muscle IR [30].

Like all flavonols, Qtn has powerful direct antioxidant effect, as a scavenger of toxic oxygen derivatives, and indirect effects, mediated by the stimulation of the Nrf2/ARE system (*B in Figure 2). In a model of IR induced in liver cells by oleic acid overload [103] Qtn increased glucose uptake (*D in Figure 2) and reduced triglyceride accumulation (*G in Figure 2). At the same time, it increased the content of cellular glutathione and antioxidant enzymes (superoxide dismutase, catalase and glutathione peroxidase) and reduced the generation of lipid peroxides. In endothelial cells, palmitate induces IR and increases the production of ROS—phenomena counteracted by Qtn and quercetin-3-O-glucuronide [123].

High-fat diets are among the most followed experimental models for inducing obesity, glucose intolerance and IR. Oxidative stress and an impaired skeletal muscle mitochondrial function may play a pivotal role in the onset of IR during diet-induced obesity. In a model of this type, regarding C57BL/6J mice fed with high fat content (45% of energy derived from them) [100], it was observed that the primary defect was the reduced ability of insulin to release glucose from the liver. In this case, adding Qtn to the diet (1.2%) for 8 weeks failed to normalize the metabolism. One possible explanation lies in the dosages. Indeed, in HFD-fed mice, a low dose of Qtn reduced IR and attenuated HFD-induced increases in fat mass and body weight [105]. Interestingly, such positive effects were not observed with a much higher dose (600 ug/mouse/day, corresponding to about 30 mg/kg). Among the biochemical parameters measured, the one most closely related to the metabolic effects of low-dose Qtn was the increase in peroxisome proliferator receptor gamma 1 alpha coactivator (PGC1α) in the muscle. PGC1α is a transcriptional coactivator that coordinates mitochondrial biogenesis and function. Of course, the results of experimental models may depend on important details, as evidenced by the fact that Qtn supplementation alters the intestinal microbiota and through this type of modification reduces inflammation in C57BL/6J mice made obese with HFD [118].

A mixture of resveratrol + Qtn has beneficial effects in oxidative stress induced by a sucrose-rich diet in rats [124]. The antioxidant properties were verified with decreased lipid peroxidation and increased catalase, superoxide dismutase, glutathione-S-transferase, glutathione reductase and overexpression of the main factor Nrf2, which increases antioxidant enzymes and GSH.

Bile duct ligation (BDL) is a surgical model performed in rodents to produce IR, accompanied by increased oxidative stress, which results in liver fibrosis. The molecular mechanism of liver injury by BDL also involves the activation of superoxide production by NADPH oxidase (NOX1) [125]. Qtn at a dose of 30 mg/kg/day significantly alleviated liver injury in BDL rats, reduced liver enzyme toxicity, and reduced mRNA and protein expression of Rac1, Rac1-GTP and NOX1 (*B in Figure 2). In the same model, the work of Khodarahmi et al. [110] demonstrated that the antidiabetic impact of Qtn was associated with increased IRS-1 and decreased NOX1 expression levels, together with down regulation of Rac1-GTP, Rac1, HIF-1alpha and ERK1. Qtn also inhibited NADPH oxidase expression or function in a rat polycystic ovary-related IR model [109], in a Dichlorodiphenyltrichloroethane (DDT) liver toxicity model [126] and in rat cardiomyocytes with T2DM induced with a high-calorie diet and streptozocin [113].

A further possible contribution of Qtn in the treatment of diabetes and IR is the inhibition of ferroptosis, a mechanism involving cellular damage related to oxidative stress in pancreatic beta cells [127].

However, the role of antioxidant supplementation in humans remains controversial, and studies have produced conflicting results on metabolic disease-related mortality [30]. Therefore, it is likely that the beneficial action of Qtn is not linked only to its capacity as a direct antioxidant. A particularly interesting action is the inhibition of the enzyme NADPH oxidase, responsible for the production of ROS during inflammatory reactions, [128] but also implicated in numerous crucial physiological processes, including cell signaling, regulation of gene expression [129] and even cardiac pathophysiology [130,131]. The inopportune activation of NADPH oxidase is part of the mechanisms that link overeating, oxidative stress and inflammation, which are positively regulated by the Mediterranean diet and by polyphenols [132].

### 4.2. Regulation of Cell Signaling Pathways

The valuable actions of Qtn in preventing or reversibilizing IR have many targets that inhibit oxidative stress and “unblock” signal transduction pathways via the protein kinase pathways described above. Dai et al. investigated the role of IR in TNF-α-induced C2C12 skeletal muscle and cell damage [104]. Phosphorylation of AMPK was significantly inhibited in treated cells, while Qtn enhanced glucose uptake in a dose-dependent manner through activation of Akt and AMP-activated (AMPK) pathways (*C and *E in Figure 2). In in vitro mixed cells, very low doses of Qtn (0.1 nM and 1 nM) significantly increase glucose uptake via translocation of the GLUT4 channel to the plasma membrane (*D in Figure 2) [111]. Qtn primarily activated the AMPK signaling pathway at lower doses, but it also activated IRS-1/PI3K/Akt signaling at 10 nM. In the same paper, oral administration of Qtn glycoside to mice at 10 and 100 mg/kg body weight significantly induced the translocation of GLUT4 to the skeletal muscle plasma membrane.

Nanomolar active doses are within the range of Qtn concentration, which has been found to be reached during therapeutic intervention trials in humans [133,134]. Despite absorption of Qtn in reportedly 9–20% of food intake, concentrations of Qtn in the blood range from 300 to 750 nmol/L after consumption of 80–100 mg of Qtn equivalent [111]. Qtn in plasma reached 431 nmol/L (0.13 μg/mL) after 1-week supplementation with 150 mg/d pure Qtn [135], 0.63 μmol/L (0.19 μg/mL) after 1-week supplementation with 80 mg/day Qtn equivalents from onions [136] and reached a maximum of 1.5 μmol/L (0.45 μg/mL) after 28 days of supplementation with high doses of Qtn (>1 g/d) [137].

In another laboratory system, myotubes from healthy donors were cultured for 24 h without and with resveratrol or Qtn to evaluate their effects on glucose metabolism, as well as the expression of key metabolic proteins and genes [33]. Both polyphenols increased insulin-stimulated glycogen synthesis and reduced lactic acid production in human myotubes. In these experiments, Qtn increased AMPK, IRS-1 and AS160 phosphorylation under basal conditions and GSK3beta under insulin-stimulated conditions (*G in Figure 2). Resveratrol tended to increase the phosphorylation rates of AMPK and GSK3beta.

Another elegant evidence of the intervention of Qtn on insulin signaling systems was offered in the model of IR established in C2C12 skeletal muscle cells by stimulation with palmitic acid (PA) [117]. A non-cytotoxic dose of Qtn has been found to promote glucose uptake and inhibit oxidative stress. Qtn inhibited the methyladenosine and METTL3 (*H in Figure 2), while it increased the protein expression of PRKD2, GLUT4 and p-Akt. Additionally, Qtn had promoter effects on superoxide dismutase (SOD), GSH. Other authors induced IR in liver cells with palmitic acid (PA) and Qtn significantly increased glucose uptake and expression of glucose transporter 2 (GLUT2) and GLUT4 [31]. A novel observation is that Qtn suppresses phosphorylation of IRS-1 on serine 612 (that is an inhibitory signal), instead it promotes phosphorylation on tyrosine and the expression of PI3K, as well as Akt and GSK3beta. Finally, the molecular docking result showed that Qtn could bind to insulin receptors, interacting with three residues, including GLU-1135, PRO-1129 and ASP-1170, in the active pocket of the receptor (*A in Figure 2). In short, the data confirm that Qtn improved the IR by increasing the signaling pathway leading to glucose uptake and glycogen production and perhaps by direct modulation of receptor sensitivity. Additionally, in the umbilical cord endothelial cell model, palmitate induced IR [123], while Qtn and quercetin-3-O-glucuronide positively regulated IRS-1 phosphorylation and restored downstream Akt/eNOS activation, leading to an insulin-mediated increase in NO level.

In the HepG2 cell model of non-alcoholic-fatty-liver disease (NAFLD) [102], Qtn enhances tyrosine phosphorylation in the insulin signaling pathway and reduces the expression levels of the protein-1c-binding sterol regulatory element (SREBP-1c) compared to the control group (*G in Figure 2). SREBP-1c is a transcription factor that is the master regulator of lipid metabolism, encoded by the sterol regulatory element binding transcription factor 1 (SREBF1) gene. SREBF1 gene variations modulate insulin sensitivity in response to fish oil supplementation [138]. Other authors reported that the beneficial effect of Qtn on glucose metabolism involves a downregulation of SREBP-1c in adipocytes [139], hepatocytes [140] and diabetic rats [141], or affected by NAFLD [142,143].

### 4.3. Regulation of Inflammation Associated with Metabolic Disorders

Even a systemic inflammatory state can favor IR [104]. Multiple biological mechanisms link inflammation to IR because signal transduction systems are vulnerable to cytokines and also to other substances such as C-reactive protein. Tumor necrosis factor causes IR by inducing transcription of inflammatory genes and directly impairing insulin signaling via the insulin receptor substrate (IRS)-1/2 [144,145]. In IR models (Table 2), the anti-inflammatory effect of Qtn is evidenced by the inhibition of the production of key cytokines and c-reactive protein.

In addition to what has already been seen about NADPH oxidase, there is much evidence that Qtn acts by regulating the production of inflammatory mediators. In studies on human basophils, very low doses of Qtn, such that they can be achieved with dietary supplementation, are able to inhibit histamine release [146,147], probably by inhibition of phosphoinositide-3 kinase-delta (PI3Kδ) [12]. There is extensive evidence that Qtn suppresses the release of pro-inflammatory markers such as IL-1beta, IL-6 and TNF-α (*F in Figure 2) [148]. Chemokines associated with macrophage M1 polarization such as CCL-2 and CXCL-10 were also effectively reduced by Qtn treatment [149].

One of the links between the 2 phenomena could be the inhibition of signaling pathways mediated through IRS-1 [150]. These authors demonstrated that C-reactive protein (hsCRP) can cause IR by increasing the phosphorylation of 2 serines, Ser(307) and Ser(612), on the IRS-1 by Jun N-terminal Kinase (JNK) and ERK1/2, respectively, leading to an inhibition of the IRS-1/PI-3K/Akt/GSK-3 pathway, and thereby, impaired translocation of GLUT4 and glucose uptake.

One of the first works to demonstrate the anti-inflammatory effect in a model of IR was that of Chuang et al. [101]. Treatment of primary cultures of newly differentiated human adipocytes with Qtn prevents TNF-α from directly activating ERK and NF-kB, which are potent inducers of gene expression of IL-6, IL-8 and MCP-1 and negative regulators of insulin signaling. Qtn prevented TNF-α-mediated serine phosphorylation of the insulin receptor substrate-1 and protein tyrosine phosphatase-1B (PTP1B) gene expression, whose importance has been highlighted in the previous section.

IR induced by oleic acid (OA) in culture medium induces fatty liver condition in HepG2 cells, increased lipid peroxidation and inhibition of glucose uptake and cell proliferation. These changes are counteracted by Qtn, with increased cell growth and increased glucose influx mediated by insulin [103]. Qtn reduced TNF-α and IL-8 by 59.74% and 41.11%, respectively, and inhibited the generation of lipid peroxides by 50.5%. Hence, Qtn effectively reversed the symptoms of NAFLD by reducing triacylglycerol accumulation, IR and inflammatory cytokine secretion in hepatocyte cells.

IR is a clinical feature of polycystic ovary syndrome (PCOS), possibly related to common factors controlling insulin receptor signaling, ovarian steroidogenesis and pituitary LH release [151]. Wang and collaborators [109] demonstrated that Qtn can reduce IR in PCOS, induced in rats with dehydroepiandrosterone administration. Qtn improved IR, reduced blood insulin levels, moderated the Toll-like/NF-kB receptor signaling pathway and inflammatory cytokines (*F in Figure 2). Additionally, Qtn increases the levels of AMPK and sirtuin (SIRT-1) in the ovarian tissue of PCOS rats [152]. Experimental studies in women with PCOS [153,154,155] and systematic reviews suggest that Qtn is able to help correct hormonal disturbances and metabolic disorders in PCOS also in humans [94,156,157].

Experimental metabolic disorders, including IR, can be induced by particulate matter (PM) administration [116]. Male C57BL/6 mice were exposed to filtered ambient air or PM for 18 weeks. Chronic exposure to PM caused inflammation in systemic and visceral white adipose tissue, with increased serum IL-6 and TNF-α levels and macrophage infiltration characterized by NLRP3 inflammasome activation. The metabolism of glucose into fat was impaired and IR occurred throughout the body. Qtn administration significantly inhibited inflammation and the NLRP3 inflammasome and ameliorated the signaling abnormalities characteristic of IR.

## 5. Effects of Silymarin on Insulin Resistance/Hyperinsulinemia and Cardiovascular Changes

Smn is a flavonoid extracted from *Silybum marianum*, composed of multiple components among which Silibinin is the most active. After oral administration, absorption is poor but improves significantly if nano particles are engineered [158]. It reaches a peak plasma concentration in 6 h and is eliminated mainly by sulfate glucuronic in the liver [159]. Among the natural substances, focused to counteract the alterations responsible for the onset of IR and its progression to T2DM and CVDs, Smn represents a safe and effective candidate [160].

### 5.1. Molecular Mechanisms of Silimarin Action on Metabolism

Oxidative stress and inflammation are key mechanisms underlying IR and its progression to diabetes and cardiovascular complications. The homeostasis of glucose and insulin is subject to attacks by oxidative and inflammatory mechanisms. Smn administration contributes to antioxidant action by different ways (*B in Figure 2): by preventing ROS generation, by scavenging free radicals, by activating vitagenes (a group of genes regulating cellular homeostasis and the response to stress), maintaining mitochondria activity, and above all, by activating a group of antioxidant enzymes and molecules to guarantee the optimal redux status of the cells. This latter is possible via transcription factors such as Nfr2 and NF-kB [161,162]. The direct scavenger’s activity is described only in the gut, compared to other biological systems, while its activity is well documented in the mitochondria chain. Smn can decrease the oxidative stress induced by chronic hyperglycemia, protecting the mitochondrial structure and function by decreasing the ROS-producing enzymes, thus reducing ROS formation. Smn increases superoxide dismutase (SOD) activity and protects from cell injury, upregulating the mitochondrial membrane potential [163,164].

As in the case of Qtn, Smn too exerts inhibitory effects on inflammation, which is considered a booster for diabetes progression. In IR, pro-inflammatory cytokines changes diminish insulin sensitivity (*F in Figure 2). The anti-inflammatory effect of Smn has been documented by inhibition of LPS-stimulated morphological changes of macrophages [165]. LPS also stimulates the MAPK activation that is inhibited by Smn treatment. The inhibition MAPK and NF-kB—other fundamental factors involved in macrophages activation and cytokine gene expression—plays a relevant action in mediating macrophage inflammatory response [166,167].

An important mediator of inflammatory response and phagocytosis is the NO, whose production is inhibited by Smn. NO production and iNOS (inducible nitric oxide synthase) gene expression inhibition in macrophages are demonstrated in vitro and in vivo in mice by Kang et al. [168]. The study, performed in Smn-treated mice and in the Raw 264.7 cell line, showed a dose-dependent and suppression of LPS induced NO production, both with a complete abrogation of iNOS mRNA expression.

Abnormal changes in cytokines can reduce insulin sensitivity in target organs. The study by Guo et al. demonstrated that Smn treatment decreased TNF-alfa, IL-6 and IL-1b in the serum of induced obese and IR mice, compared to untreated controls [169]. At the same time, insulin sensitivity was significantly improved in the treated group, in fact the elevation of fasting insulin was significantly reversed, and the insulin tolerance test (ITT) improved. An additional protection has been demonstrated in vitro through the activation of vitagenes, which synthetize for heat shock proteins, sirtuins and thioredoxine a recognized protective molecule against oxidative stress [170].

Another important antioxidant action exerted by Smn in the regulation of glucose homeostasis is the elevation of sirtuin-1 (Sirt-1) expression, demonstrated by Feng et al. [171]. Sirt-1 plays a key role-in the development of IR; it not only regulates glucose-dependent insulin secretion but also stimulates insulin pathway signaling in insulin-sensitive organs. The regulation of adiponectine is mediated by enhancing encoding genes that control secretion from adipocytes [172]. The up regulation of adiponectine improves IR.

Part of the insulin sensitizer action, mediated by Sirt-1, is exerted by PPARγ, whose regulation leads to low blood glucose levels and improved metabolism. PPARγ controls a gene network involved in glucose homeostasis, like the GLUT-4 transporter, together with numerous molecules, such as resistine, leptine, TNF-ɑ, etc., implicated in insulin sensitivity [173,174,175,176]. The action of Smn on GLUT-4 is mediated not only by PPARγ but also by the activation of the IRS1/PI3K/Akt pathway that increases the glucose uptake and, consequently, the GLUT-4 translocation [177]. In this sense, the action of Smn and Qtn appears fully synergistic (*D in Figure 2).

As described above, pancreatic β cells synthetize, store and release insulin to maintain glucose homeostasis and its disfunction leads to IR and ultimately to diabetes. Bouderba et al. demonstrated that administration of Smn, in a murine model of obesity and diabetes resembling human diabetes, restored antioxidant status—namely, decline of glutathione, rise of peroxidation and augmented levels of SOD, therefore reducing IR, diabetes and hepatic steatosis [178]. Pancreas activity has been shown to depend on the transcription of the NKx6.1 (NK6 homebox1) gene. It is fundamental for both the differentiation and maintenance of pancreatic cells. NKx6.1 transcription is activated by pancreatic and duodenal homebox 1 (Pdx1), a transcriptional factor active during the differentiation and maturity of βcells, showing their cooperation to maintain pancreas activity. Soto et al. demonstrated that Smn can restore pancreas function and morphology after alloxan-induced damage in Wistar rats. Smn administration led to normal insulin and glucose concentration and restored Pdx1 mRNA expression with respect to controls, thus recovering pancreas function [179]. The same group later showed that treating partially pancreatomizated rats with Smn could increase Nkx6.1 and augment the number of βcells, raising the insulin genic expression compared to untreated animals. The treated mice showed significant lower serum glucose levels and increased insulin concentrations, comparable with unpancreatomizated controls [180].

Another interesting action of Smn in βcells is the suppression of iNOS by modulating NF-KB activity and extracellular signal regulated proteins (ERK 1 and 2), thus preventing beta-cell disruption [181]. Furthermore, Smn significantly increases the GSH/GSSG ratio, both in the plasma and the pancreas, preventing lipid peroxidation and hyperglycemia in alloxan-treated rats [46].

Visceral obesity is associated with insulin resistance and hyperinsulinemia, and Smn can reduce IR by reducing visceral fat. The hepatic steatosis activates gluconeogenesis interfering with insulin-stimulated tyrosine phosphorylation of IRS 1 and IRS2, thus inhibiting the glycogen synthase. As demonstrated by Samuel et al., IR can be determined by hepatic lipid accumulation, without a contextual peripheral organ IR or fat deposition [182]. Smn reduces IR by inhibiting hepatic gluconeogenesis through the down regulation of glucose 6 phoshatase (G6Pase) and phoshoenolpyruvate carboxykinase (PEPCK), the key enzymes of this metabolic pathway [183,184].

The reduction of glucose production was demonstrated also in the study of Yao et al., where the treatment with Smn in a group of high-fat diet Sprague Dawley rats led to reduction of visceral fat by enhancing lipolysis, and a decrease of HOMA- IR and ITT, compared to rats with the same food regimen but without Smn [185].

### 5.2. Clinical Studies of Silymarin for Insulin Resistance

The above documented pharmacological activity has suggested Smn as a good candidate for treatment of IR, T2DM and their complications. Some clinical studies have been also conducted to assess whether these biological actions can translate into a healthy improvement. Elgarf et al. have demonstrated, in a randomized controlled study, that the adjunct of 140 mg of Smn, three times a day, to standard anti-diabetic therapy leads to an improvement of glycemic indices in diabetic patients [186]. After three months of treatment there was a highly significant improvement in fasting glucose (FG), glycated hemoglobin (Hba1c), fasting insulin (FI) and HOMA index in the group treated with Smn. These results were later confirmed by an Iranian group. In this case too, a significant improvement of all studied glycemic indices was observed after 45 days of treatment with Smn, compared to controls [187].

A more recent observational study, conducted in 200 newly diagnosed T2DM patients, has shown that the adjunct of 200 mg of Smn bid to standard therapy, significantly decreased FG, Hb1c, HOMA IR and FI after 3 months of treatment, thus confirming the results of previous randomized clinical trials (RCTs) [188].

Two recent meta-analyses have been conducted to assess the results of Smn administration to T2DM patients in RCTs. In the first, 7 studies including 350 patients were analyzed. All the glycemic indices were improved, while the lipid profile failed to lead to a solid conclusion [189]. A larger meta-analysis of 16 studies, consisting of 1358 patients, concluded that Smn addition to standard therapy reduced FG, HOMA-IR, Hba1c and lipid profile compared to controls [190].

Despite discordant results regarding the lipid profile, all the examined studies seem to agree on the safety and efficacy of Smn in the treatment of IR and T2DM. The review of the scientific literature, and what is reported above, shows good pharmacological activity of Smn against IR/Hyperin, suggesting this natural substance as a good candidate for treatment of IR, T2DM and their complications.

### 5.3. Effects of Silymarin on the Cardiovascular System

Although not conclusive, particularly regarding clinical studies, there is ample scientific literature on the protective effects of silymarin against the processes of atherosclerosis and the development and progression of cardiovascular diseases. On the other hand, numerous studies have highlighted the beneficial antioxidant and anti-inflammatory actions of silymarin, and its positive action on endothelial dysfunction [167,170,191]. Endothelial dysfunction, as is well known, is a pathogenetic key event in the development of cardiovascular diseases in subjects with insulin resistance/hyperinsulinemia and/or diabetes. Underlying the endothelial dysfunction is an increase in asymmetrical dimethylarginine (ADMA), an inhibitor of nitric oxide synthase (NOS), which results in a reduction in the formation of nitric oxide. A study in db/db mice showed that the administration of Smn for 4 weeks resulted in a reduction of the endothelial dysfunction by reducing the circulatory and vascular levels of ADMA and consequent reduction of NOS inhibition [191].

The positive effect of Smn on endothelial dysfunction was subsequently confirmed in a study to test the effect of Smn on the vascular function of older rats [192]. Aging unfortunately involves the appearance of endothelial dysfunction with the thickening and hardening of the vascular wall, which are among the main causes of cardiovascular disease. In this study, aorta rings from rats aged 22 months were incubated in baths with Smn, Smn/L-nitroarginine methyl ester or Smn/indomethacin and compared with those from rats 3–4 months old. Aging increased sensitivity to phenylephrine and decreased contraction to KCL, while Smn improved both. Aging also deteriorated the relaxation caused by acetylcholine, whereas Smn improved its response. L-nitroarginine methyl ester prevented the effect of Smn. Thus, Smn improved endothelial dysfunction and vascular tone alteration caused by aging and this was due to a prevalent action on the nitric oxide pathway.

Cellular oxidative stress determines the release of free radicals, toxic at vascular level, from the endothelial cells and the VSMC that interact with the components of the same cells (DNA, proteins, lipids) determining cardiovascular damage [193]. A recent review article of the scientific literature on the subject has highlighted how Smn has important antioxidant activities also in favor of the cardiovascular system cells and offers protection against atherosclerosis, arterial hypertension and cardiotoxicity induced by oxidative stress [194,195].

The accumulation of ROS in the vascular wall produces LDL oxidation, which in turn promotes the formation of the lipidic plaque. One important mechanism underlying the beneficial effects of Smn against atherosclerosis is that it reduces LDL oxidation, a significant step in the development of the atherosclerotic process [196], through its relevant antioxidant properties [170]. Nephrotoxicity in insulin resistance/hyperinsulinemia states and in type 2 diabetes mellitus is mainly caused by oxidative stress. Experimental studies have shown that Smn can have beneficial protective effects on the development of diabetic nephropathy [197]. In this regard, of particular interest is a manuscript reporting the results of a study testing the effects of Smn on the activity and gene expression of three enzymes belonging to the category of oxidoreductases (superoxide dismutase, glutathione peroxidase, catalase) and on renal tissue damage in rats with alloxan-induced diabetes mellitus [198]. The administration of alloxan caused a significant reduction in the activity and gene expression of all three enzymes. Twenty days after intoxication with alloxan, a sample of rats was treated for nine weeks with Smn. In the treated rats, Smn prevented tissue damage and significantly improved activity levels and gene expression of the said three enzymes. Another interesting article demonstrates the beneficial effects of Smn on another vascular district, that of the retina. In fact, this study shows that Smn can also be useful in preventing diabetic-induced hyperpermeability in human endothelial cells of the retina [199].

## 6. Indirect Effects Related to the Microbiota

The gut microbiota plays a pivotal role on the metabolism of carbohydrates, proteins, vitamins, bile and polyphenols [200]. A disordered diet and excess fat can cause imbalances in the intestinal microbiota, with increased permeability of the mucosa and passage of pro-inflammatory compounds, such as lipopolysaccharide (LPS), into the lymph and blood. LPS can increase inflammatory processes by synergizing with cytokines and promoting metabolic syndrome [108,201]. Polyphenols such as curcumin, Qtn and catechins also improve metabolic syndromes such as diabetes, obesity and hypertension through intestinal microbiota (*I in Figure 2) [202,203,204].

It has been recently reported that one potential mechanism at the base of the beneficial effects of Bbr on the lipid metabolism and IR could be related to its effect on gastrointestinal microbiota [85]. Gut microbiota can synthesize a group of substances important to maintain physiological body functions (trimethylamine, short-chain fatty acids, bile acids, etc.). Bbr regulates the synthesis of these substances by balancing the amount of gut bacteria deputed to their synthesis [205]. It has been shown that Bbr treatment has beneficial effects in obese rats with high-fat diets. In fact, in this study, an 8-week treatment with Bbr reduced fasting glycemia and insulin resistance, and corrected gut microbiota composition altered by the high-fat diet, which in turn produced an increased release of lipopolysaccharides (LPS) in the plasma. Therefore, Bbr treatment can reduce insulin resistance, at least in part, by a correction of the gut microbiota and by inhibiting LPS/Tool Like Receptor 4 (TLR4)/TNF alfa signaling in the liver [206].

The intestinal microbiota transforms the polyphenols into their metabolites to make them bioactive and at the same time the latter modulate the microbiota. Qtn has a profound influence on the intestinal microbiota, which in turn modulates its bioavailability [207]. Male C57BL/6J mice were fed a high-fat diet supplemented with 0.05% Qtn for 6 weeks and the researchers also checked the gut microbiota [112]. Mice whose diet was supplemented with Qtn gained less body weight, liver and fat compared to the Qtn-free fatty diet, while liver lipid and blood glucose levels were also lowered. In the stools, Qtn supplementation significantly increased the relative abundance of *Akkermansia* and decreased the Firmicutes/Bacteroidetes ratio. In studies in Wistar rats, the addition of Qtn to a diet high in fat and sugar prevented body weight gain, IR and hyperinsulinemia [107]. The integration of Qtn also rebalanced the intestinal microbiota by inhibiting the growth of bacterial species *Erysipelotrichaceae*, *Bacillus* and *Eubacterium cylindroides*, which were instead increased by an incorrect diet.

Porras et al. have thoroughly investigated the effects of Qtn on intestinal health and the correlated IR [108]. In a C57BL/6J mouse model, a high-fat diet (HFD) for 16 weeks induced metabolic syndrome, fatty liver disease and IR. The microbiota, too, was altered, with an increase in the Firmicutes/Bacteroidetes ratio, an increase in Gram-negative bacteria and also in the genus Helicobacter. Qtn supplementation (0.05% (*wt*/*wt*)) reduced IR and the accumulation of intrahepatic lipids and lipoperoxidation. Qtn also restored the gut microbiota and reduced the endotoxemia-mediated TLR-4 pathway. The prebiotic capabilities of Qtn were confirmed in a subsequent work by the same group [208], suggesting that the metabolic syndrome could find a complementary therapeutic approach in the modulation of the intestinal microbiota.

Even more recently [118], it was confirmed that in mice on a high-fat diet (HFD) rendered obese, Qtn supplementation improves glucose tolerance and also improves the gut microbiota, reducing inflammation of the gut adipose tissue. Mice were administered 50 mg per kg of body weight of Qtn by gavage for 20 weeks. Qtn recovers the intestinal barrier function and regulates the intestinal microbiota such as *Adlercreutzia*, *Allobaculum*, *Coprococcus*, *Lactococcus* and *Akkermansia*.

Short-chain fatty acids are fatty acids with fewer than six carbon atoms (C), produced when beneficial bacteria in the gut ferment certain nutrients, and are the main source of energy for the cells in the wall. Short-chain fatty acids are intestinal anti-inflammatory agents also involved in the metabolization of important nutrients, such as carbohydrates and fats. In a rat model of loperamide-induced constipation, Qtn (25 mg/kg and 50 mg/kg) improved intestinal peristalsis and short-chain fatty acid (SCFA) concentration by increasing levels of multiple signaling pathways in intestinal cells [209].

Recently, Lee et al. demonstrated that Smn has a protective effect against liver steatosis and obesity in a group of mice feed with a gavage of HFD for 8 weeks [210]. In the treated group gut, the microbioma composition changed significantly, in turn improving NAFLD. These results were accompanied by major changes in cytotoxic metabolites that decreased. Another interesting study showed that treatment with Smn increased the richness of gut bacterial species in obese HFD mice with reduced microbiota diversity [211]. The authors hypothesized that the antioxidant activity could improve the bacterial B12 biosynthesis by regulating environmental redox potential.

## 7. Conclusions and Prospects

Obesity is unfortunately on the rise worldwide (World Health Organization, World Obesity Day 2022–Accelerating Action to Stop Obesity, 4 March 2022). Obesity and, more specifically, visceral obesity are frequently associated with IR/Hyperin [212]. However, IR/Hyperin may also be present in normal weight or, even, lean subjects [6,213]. Being strictly correlated with diabetes, endothelial dysfunction and hypertension (see Figure 1), IR/Hyperin should be considered a risk factor for cardiovascular disease and, as such, should be subject to early screening in suspected subjects and treated early, as in the case of other cardiovascular risk factors.

There is extensive scientific literature supporting the fact that IR/Hyperin should also be considered as an independent risk factor for the development of cardiovascular diseases [3,214,215]. However, since IR is associated with other risk factors, it is difficult to distinguish whether IR/Hyperin is a risk factor per se, or whether it facilitates the development of cardiovascular diseases and the consequent associated increase of cardiovascular mortality because it determines the development of other risk factors [216,217]. This is probably a moot point, as it is very likely that both hypotheses are true; namely, that IR/Hyperin has directly negative actions in the development of cardiovascular abnormalities and that it can lead to other risk factors contributing to its negative actions. What is certain is that—despite the intervention of other cardiovascular risk factors, first being dyslipidaemias—diabetes, arterial hypertension and mortality from cardiovascular events are still too high (https://www.who.int/health-topics/cardiovascular-diseases#tab=tab_1, accessed on 18 April 2023). This could be due, at least in part, to the fact that there were no appropriate incisive interventions in the early detection and treatment of IR/Hyperin.

In fact, hyperinsulinemia, which is the main marker of IR, can go unrecognized for a long time and damage the cardiovascular system over the years if no action is taken [15]. The fact that hyperinsulinemia can determine cardiovascular alterations over time has been widely discussed [216,217], and it has also been verified that the use of drugs that increase insulin sensitivity, in the treatment of type 2 diabetes mellitus, can reduce the possibility of cardiovascular death compared to insulin therapy alone [3]. However, there was unfortunately little interest in pointing out that an excess of insulin treatment could become harmful, although it was shown that the use of intensive insulin therapy for 3.5 years, in order to bring glycated haemoglobin to normal range values, increased mortality and did not significantly reduce major cardiovascular events compared to the standard therapy [45,218].

Insulin, as it is well known, is a hormone and, like any hormone, it causes harmful effects both when it is low and when, as in the case of IR, it is chronically increased for a long time. In particular, in our review we highlighted the deleterious effects of IR/Hyperins on the cardiovascular system and discussed the possibility of a prompt intervention with three natural safe substances (Bbr, Qtn, Smn), which have potential synergistic mechanisms in the treatment of this pathology. The prevention and rectification of IR/Hyperins, in the light of the demonstrated pathophysiological mechanisms by which it causes damage to health and progression toward cardiovascular diseases, must be recognized as soon as possible, as we do for other more well-known risk factors (e.g., arterial hypertension and hypercholesterolemia), and promptly treated. Since there are not pharmacological-therapy-approved natural substances that have clearly shown to interfere with the harmful pathophysiological mechanisms of IR/Hyperins, they could represent an effective alternative. It is therefore essential to reduce IR to reduce chronically increased levels of circulating insulin.

In short, considerable evidence obtained from animal experiments demonstrates that Bbr, Qtn and Smn have beneficial effects on the main mechanisms which, once dysregulated, lead to the development of diabetes mellitus, and ultimately, to the most common cardiovascular diseases. These substances can promote insulin secretion, improve IR, lower blood lipid levels, inhibit inflammation and oxidative stress, relieve hepatic lipid accumulation and regulate gut microbiota disorders in animal models [119]. Notwithstanding promising epidemiologic and observational studies, human clinical trials on the effects of the described compounds in diabetes, metabolic syndrome and cardiovascular pathology remain scarce. More clinical studies are needed in order to explore the different doses with sufficiently large samples, longer durations and to verify its real efficacy in human subjects, possibly in synergy with other natural compounds or conventional drugs. Certainly, while the molecular mechanisms of the effects of the molecules considered on animal and cellular models are now clear, there remains a significant knowledge gap for their clinical applications. In particular, the effective doses of the individual compounds should be investigated, the possible synergisms (which it is hoped will serve to reduce the doses, and therefore, the potential side effects) and the timing of administration, as well as the interactions with other drugs potentially used by patients who have glucose metabolism disorders and/or cardiovascular diseases.

The extensive scientific literature demonstrates that many natural substances can act on IR by reducing it. In particular, experimental in vitro and in vivo studies in animals, as discussed in this review, and clinical studies in humans show the important benefits of berberine, Qtn and silymarin on IR and circulating insulin levels, acting on the different pathophysiological mechanisms underlying this dysfunction [8,9,51,53,58,77,121,177,185,188,190,219,220,221,222]. In addition to the actions already described, Smn inhibits glycoprotein P as well at the membrane level of the enterocytes [223]. Glycoprotein P is the best-known component of a family of transport proteins called ABCs, which use the energy from hydrolysis of ATP to transport molecules across the cell membrane. Bbr, unfortunately, is a substrate of glycoprotein P, so its absorption in the intestine would be very variable without the intervention of Smn, which has an inhibitory action on glycoprotein P. Therefore, in addition to taking advantage of any positive antiatherosclerotic and metabolic activity of silymarin, this association primarily aims to improve and increase constancy with regard to the absorption of berberine, which features a broader and more conclusive scientific literature on the topic of the treatment of IR/Hyperin and the prevention of cardiovascular abnormalities.

Considering the multiple pleiotropic and redundant biochemical actions of Bbr, Qtn and Smn, the combination of these substances in the treatment of IR could determine a synergistic mechanism enhancing the effectiveness and consequent possibility of reducing the dosages of individual substances, to improve maximum tolerability and allow nutraceutical dosages. This hypothesis should, of course, be verified through clinical studies with sufficient numbers of subjects—randomized, double blind and placebo controlled.

The first line in the treatment of IR/Hyperin should consistently be the change of both dietary and physical lifestyle, although it has been demonstrated that only a more intensive and constant dietary and exercise program was able to produce significant improvement of insulin sensitivity, unlike a modest or inconstant program [224]; however, it has been extensively verified in clinical practice that this is well implemented only by a minority of subjects, which means that an aid becomes necessary in most cases. The use of these natural substances could be a choice; in fact, they would certainly be better accepted—compared to a drug-based therapy—by subjects who know they have the problem but are asymptomatic or paucisymptomatic.

This hypothesis, once verified, could be a solution, at least partly, to the problem of IR/Hyperin, understood as a cardiovascular risk factor. In this case, it should be mandatory to make an early diagnosis in subjects identified as potentially insulin resistant and an early treatment with effective natural substances in all those subjects who have not responded sufficiently well to a lifestyle change. In our opinion, together with all the possible limits, this could significantly contribute to preventing cardiovascular diseases, which, despite efforts made, are still the leading cause of death in developed and developing countries.

## Figures and Tables

**Table 1:** Number of bibliographic citations in PubMed-NLB for keywords concerning insulin resistance (IR) and the indicated natural compounds, searched with EndNote (ClarivateTM) on 8 May 2023.

Compound	Position of Key-Word Compound	Insulin Resistance in Title	Insulin Resistance in Abstract
Berberine	Title	53	192
	Abstract	62	268
Quercetin	Title	19	100
	Abstract	38	244
Silymarin	Title	6	31
	Abstract	15	68
Polyphenols	Title	28	139
	Abstract	98	591
Resveratrol	Title	60	281
	Abstract	82	467
Catechin	Title	1	9
	Abstract	38	189

**Table 2:** Summary of experimental tests of the effects of Qtn on insulin resistance parameters.

Model	Dose of Qtn	Main Effects	Ref.
IR induced by high-fat diet (mouse)	1.5% (*wt*/*wt*) in diet for 8 weeks	No improvement	[100]
IR induced by TNFα in primary human adipocytes	10–30 µmol/L in vitro	↓ NF-kB and cytokine secretion ↓ PTP1B gene expression	[101]
NAFLD model and IR induced by fatty acids in hepatic HepG2 cell line culture	0.1–100 µmol/L in vitro	↑ phosphorylation of insulin-signaling pathway (IRβ e IRS-1) ↓ sterol regulatory element-binding protein-1c (SREBP-1c) and fatty acid synthase (FAS)	[102]
Steatosis-like phenotype and IR induced by oleic acid in hepatic HepG2 cell line culture	1–10 µmol/L in vitro	↑ insulin mediated glucose uptake ↑ glutathione content ↓TNF-α, IL-8 and lipid peroxides	[103]
IR in skeletal muscle cells treated with TNFα	10–20 µmol/L in vitro	↑ glucose absorption ↑ MAPK e Akt (PKB) phosphorylation ↓ NF-kB and INOS	[104]
IR induced by high-fat diet (mouse)	50 µg/die/mouse (low dose) or 600 µg/die/mouse (high dose) for 8 weeks	↓ IR (Low dose only). ↑ Peroxisome proliferator-activated receptor gamma coactivator 1-alpha (PGC1α)	[105]
IR induced by high-fat and high-sugar diet (rat)	30 mg/kg for 6 weeks	↓ fruttosamine, basal glycemia, insulin e HOMA-IR No differences in free fatty acid concentrations and obesity indices.	[106]
IR and obesity induced by high-fat and high-sugar diet (rat)	Added to diet 30 mg/kg/die	Prevents IR ↓ body weight Attenuates intestinal dysbiosis	[107]
High-fat diet induced metabolic syndrome (mouse)	Added to diet 0.05% (*wt*/*wt*) for 16 weeks	↓ IR ↓ lipoperoxidation ↓ LPS-mediated inflammation Restores microbiota (Firmicutes/Bacteroidetes equilibrium)	[108]
IR in dehydroepiandrosterone-induced polycystic ovary syndrome (rat)	100 mg/kg for 28 days	Recovery of the estrous cycle ↓ insulinemia ↓ TLR4, NF-kB and IL-1beta ↓ expression of p22phox (NADPH oxidase)	[109]
IR and liver fibrosis induced by bile duct ligation (BDL) (rat)	30 mg/kg/day for 4 weeks after operation	Antidiabetic effect: ↓ STAT3 e SOCS3, with ↑ IRS-1. Antifibrotic effect: ↓ Rac1-GTP, Rac1, HIF-1alpha, NOX1 and others	[110]
Myotubes L6 in vitro	1–10 µmol/L	↑ increased translocation of GLUT4 and glucose uptake ↑IRS-1/PI3K/Akt reporting	[111]
Normal ICR mice	10–100 mg/kg (quercetin-3-O-β-glucoside)	↑ increase of GLUT4 in skeletal muscle	[111]
C57BL/6J mice fed high-fat diet	0.05% in the diet for 6 weeks	↓ hyperglycemia, obesity and steatosis ↓ insulin and leptin ↑ Akkermansia and Bacteroidetes/Firmicutes ratio in feces ↓ expression of Srebf1, Ppara, Cyp51, Scd1 and Fasn genes	[112]
T2DM induced with high-calorie diet and streptozotocin (rat)	10–50 mg/kg for 8 weeks	↑ insulin sensitivity ↓ oxidative stress in cardiac mitochondria ↓ NADPH oxidase and xanthine oxidase ↑ superoxide dismutase, glutathione peroxidase, glutathione reductase	[113]
Myotubes from healthy donors	10 μMol/L (in vitro)	↑ MAPK, IRS-1, and AS160 phosphorylation in basal conditions and ↑ glycogen synthase kinase 3 (GSK3beta) in insulin-stimulated conditions	[33]
IR and inflammation induced by 60% fructose diet (rat)	100 mg/kg for 6 weeks	↑ glucose tolerance ↓ adipose tissue ↓ NLRP3 inflammasome ↓ IL-1β and IL-18	[114]
Metabolic syndrome and IR induced by 20% fructose (rat)	15 mg/kg/die	↓ glycemia and insulinemia ↓ systolic arterial pressure, triglycerides, cholesterol VLDL	[115]
IR induced by chronic exposure to PM2.5, with elevation of serum IL-6 and TNF-α and activation of NLRP3 (mouse).	50–100 mg/kg for 18 weeks	↓ glycemia and IR ↓ systemic inflammation ↓ NLRP3 in adipocytes	[116]
IR induced by high-fat diet (mouse)	10 mg/kg (gavage) for 10 weeks	↓ glycemia and IR ↓ ROS production ↑ SOD e GSH	[117]
IR induced in C2C12 myocytes by palmitic acid (PA)	5–10 µmol/L	↓ methyladenosine (m6A), METTL3 and p-IRS-1 ↑ PRKD2, GLUT4 e p-Akt expression ↓ oxidative stress	[117]
IR and obesity induced by HFD (mouse)	50 mg/kg for 20 weeks	↓ Inflammation of adipose tissue ↑ glucose tolerance Changes in gut microbiota	[118]
IR induced in HepG2 hepatic cells by PA	4–8 µmol/L	↑ GLUT4 and glucose uptake ↑ glycogen production ↓ Ser612 phosphorylation of IRS-1	[31]

↑ means increased, ↓ means decreased.

## Data Availability

Data sharing not applicable.
